# A single copy transgenic mutant FUS strain reproduces age-dependent ALS phenotypes in *C. elegans*

**DOI:** 10.17912/micropub.biology.000473

**Published:** 2021-09-22

**Authors:** Audrey Labarre, Gilles Tossing, Claudia Maios, James J Doyle, J Alex Parker

**Affiliations:** 1 Centre de Recherche du Centre Hospitalier de l'Université de Montréal (CRCHUM), Montreal, Canada.; 2 Department of Neuroscience, Université de Montréal, Montreal, Canada.; 3 Division of Experimental Medicine, Faculty of Medicine, McGill University, Montreal, Canada.; 4 Metabolic Disorders and Complications, Research Institute of the McGill University Health Centre , Montreal, Canada

## Abstract

Mutations in the human DNA/RNA binding protein FUS are associated with amyotrophic lateral sclerosis and frontotemporal lobar degeneration, including some aggressive and juvenile onset forms. Cytoplasmic inclusions of human FUS proteins are observed in various neurodegenerative disorders, such as Huntington’s disease or spinocerebellar ataxia, suggesting that FUS proteinopathy may be a key player in neurodegeneration. To better understand the pathogenic mechanisms of FUS, we created single copy transgenic *Caenorhabditis elegans *strains expressing full-length, untagged human FUS in the worm’s GABAergic neurons. These transgenic worms expressing human mutant FUS (mFUS) display the same ALS-associated phenotypes than our previous multiple copy transgenic model, including adult-onset age-dependent loss of motility, progressive paralysis and GABAergic neurodegeneration. These phenotypes are distinct from the transgenic worms expressing human wild-type FUS (wtFUS). We introduce here our *C. elegans* single copy transgenic for human mutant FUS motor neuron toxicity that may be used for rapid genetic and pharmacological suppressor screening.

**Figure 1. Expression of single copy transgenic human mutant FUS reproduces age-dependent ALS phenotypes in C. elegans:**
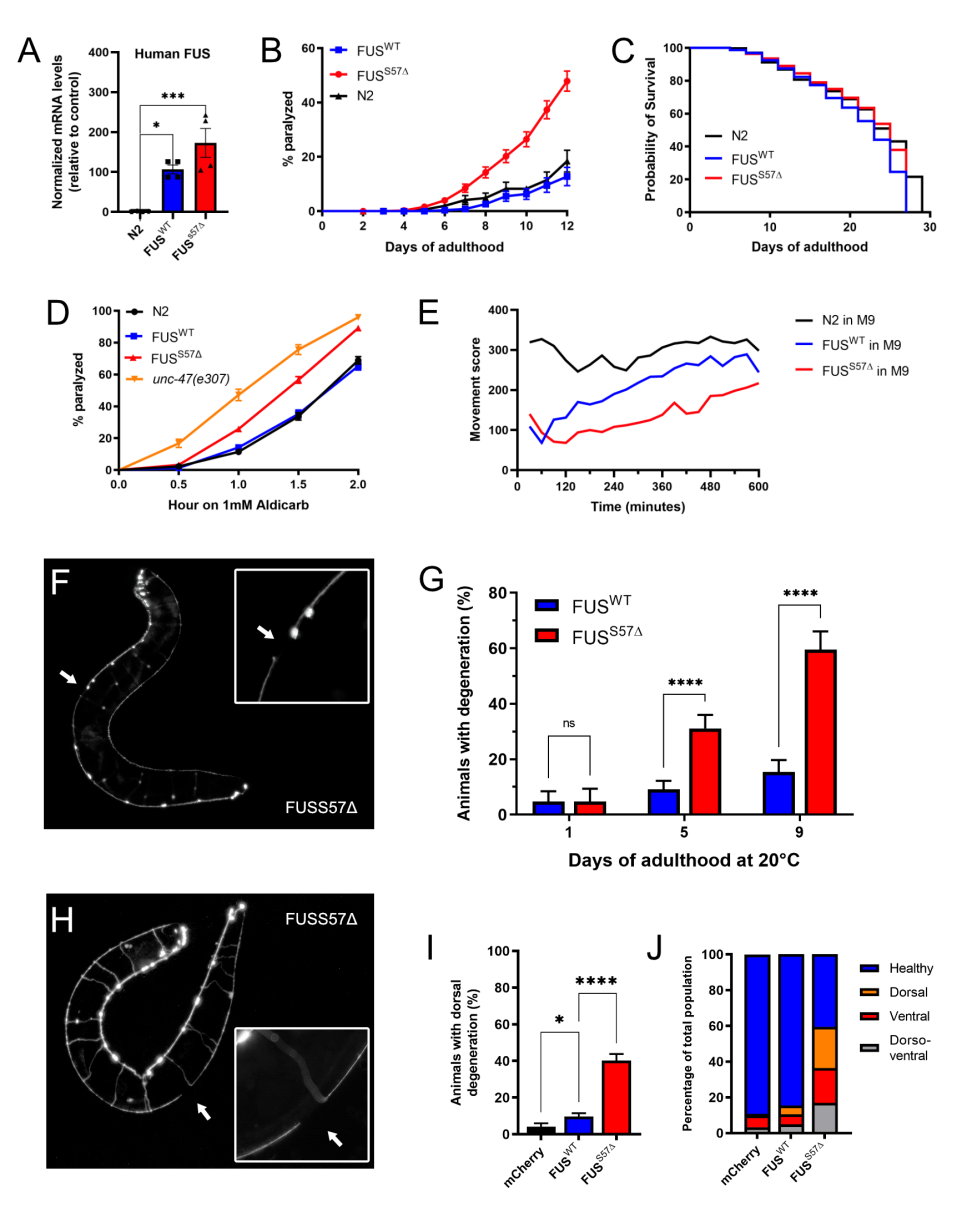
**(A)** Validation ofhuman FUStransgene expression by qPCR (taqman). **B)** Adult worms were scored daily on solid media for paralysis phenotype. Transgenic mFUS worms show increased progressive paralysis over 12 days compared to transgenic wtFUS worms (p< 0.0001). **(C)** Expression of mFUS does not affect lifespan compared to wtFUS expression. **(D)** Synaptic cholinergic transmission has been evaluated by exposing day 1 adult worms to the cholinesterase inhibitor aldicarb. Worms were scored over 4 hours for aldicarb induced paralysis. Day 1 adult mFUS worms were hypersensitive to aldicarb treatment compared to either wtFUS or N2 (p< 0.0001). **(E)** Motility was further assessed in liquid M9 media by using the PhylumTech WMicrotrackerOne instrument. Adult day 1 mFUS worms have decreased motility capacity in liquid media over 10 hours compared to wtFUS and N2 (p< 0.0001). **(F)** Representative photo of an adult day 9 old *unc-47p::mCherry;FUS* worm with a degeneration event in the ventral neuronal processes of the GABAergic motor neurons (Highlighted in the magnified frame). **(G)** Quantification of neurodegeneration in transgenic worms at days 1, 5 and 9 of adulthood. At day 5 and 9 of adulthood mFUS worms show significantly higher neurodegeneration than wtFUS worms (p< 0.0001). **(H)** Representative photo of an adult day 9 old *unc-47p::mCherry;mtFUS* worm with a dorsal degeneration event in the axon of the GABAergic motor ­neurons (Highlighted in the magnified frame). **(I)** Quantification of dorsal axonal degeneration events in adult day 9 worms. Dorsal axonal degeneration can be observed more frequently in mFUS worms compared to wtFUS or *unc-47p::mCherry* control worms (p< 0.0001). **(J)** Representation of GABAergic neuronal health in the transgenic FUS populations. mFUS worms have a higher percentage of different degenerative events in the day 9 of adulthood (Ventral, dorsal or both ventro-dorsal degeneration).

## Description

Amyotrophic lateral sclerosis (ALS) is a fatal and incurable adult-onset neurodegenerative disease characterized by the progressive loss of motor neurons leading to muscular atrophy (Vucic *et al.*, 2014). Despite the fact that the vast majority of ALS cases are sporadic, around 10% of patients have an inherited form of the disease (Renton *et al.*, 2014). Among the genes known to be involved in familial cases of the disease, Fused in Sarcoma (FUS, also called TLS) is known to be accountable for aggressive and juvenile form of ALS (Conte *et al.*, 2012; Shang & Huang, 2016). Our laboratory has previously generated multiple-copy transgenic animals expressing human wild-type and mutant full-length FUS which reproduced many aspects of the human disease (Vaccaro *et al.*, 2012). However, advances in genetic manipulation have made it possible to generate single-copy transgenic *C. elegans* animals, which can be more accurate for investigating the mechanisms of human FUS-induced neuronal death since the transgenic expression levels resemble endogenous levels. Here, we present the characterization of the first single-copy transgenic human FUS nematodes. Animals expressing human mutant FUS (mFUS) recapitulate many aspects of the human disease, including adult-onset paralysis and motor neuron degeneration. Importantly, worms expressing single copy human wild-type FUS (wtFUS) do not display these phenotypes associated with the disease. Given the many advantages of small animal models in biomedical research, we believe this new model will be a powerful tool for future chemical suppression screening.

Transgenes of full-length wild type human FUS and full-length human mutant FUS with the clinical mutation S57Δ were used for the generation of the strains. The S57Δ mutation was identified in ALS patients and is located in the N-terminus region of the FUS protein. (Belzil *et al.*, 2009). Being a motor neuron disease, we expressed human wild-type and mutant FUS in the worms’ GABAergic motor neurons, under the GABA transporter (*unc-47*) promoter (McIntire *et al.*, 1997). Transgenic lines were obtained with stably-integrated transgenes using the mos1-mediated single copy insertion (mosSCI) (Frøkjær-Jensen *et al.*, 2012). The expression of both wild-type and mutant human FUS have been confirmed by qPCR (Figure 1A).

Transgenic strains were morphologically normal and displayed no developmental defects. Although, after the first few days of adulthood, worms expressing mFUS displayed abnormal movement that progressed into paralysis. This effect was age-dependent and reached about 60% after 12 days on plates, compared to 100% in our previous multiple-copy model (Figure 1B) (Vaccaro *et al.*, 2012). About 20% of worms expressing wtFUS were affected at day 12, which showed rates comparable to non-transgenic, N2 worms (Figure 1B). We observed that mFUS and wtFUS worms had lifespans indistinguishable from non-transgenic N2 worms (Figure 1C). This suggests that paralysis is due to the expression of ALS-linked S57∆ FUS in the motor neurons and not to a general decrease in health.

The expression of mFUS in the worms’ GABAergic motor neurons results in an age-dependent paralysis, which suggests that there may be neuronal dysfunction and degeneration in these animals. To investigate we first exposed worms to the acetylcholinesterase inhibitor, aldicarb. This compound causes an accumulation of acetylcholine in the neuromuscular junctions resulting in muscular hypercontraction and acute paralysis (Mahoney *et al.*, 2006). We observed that mFUS worms were hypersensitive to aldicarb treatment, as was evidenced by their increased rate of paralysis compared to wtFUS and N2 (Figure 1D). However, mFUS worm sensitivity to aldicarb is comparable to *unc-47(e307)* mutants. These animals lack the vesicular GABA transporter gene, required for GABA transmission, and known to be hypersensitive to aldicarb-induced paralysis (Vashlishan *et al.*, 2008). There was no difference in sensitivity between non-transgenic and wtFUS worms. The hypersensitivity to aldicarb seen in the S57∆ mFUS animals suggests either reduced GABA signaling or an increase in acetylcholine secretion. Since the expression of the transgenes are driven in the GABAergic motor neurons, we believe that the presence of mFUS causes dysfunction in the GABAergic system.

The ultimate goal of developing this transgenic animal model is to use it as a tool for drug screening and genetic suppressor screening. We show that animals that express the mFUS protein display severe neuronal phenotypes that resemble human ALS, the most evident of which is a progressive, age-dependent paralysis on solid media that begins as of day 7. However, for pharmacological or genetic suppressor screening purposes this timeline is inconvenient. Our laboratory previously used a worm tracking system (wMicroTracker; Phylum Tech) and demonstrated that motility defects can be enhance from few days to few hours in liquid culture (Schmeisser *et al.*, 2017). We sought to test whether we could accelerate the onset of the paralysis phenotype in our day 1 single-copy mFUS adult worms in liquid media. We placed worms in 96-well plates in M9 Buffer into a PhylumTech wMicroTrackerOne instrument which allowed us to quantify in an unbiased manner worm swimming behavior through light scattering. The analysis confirmed that mFUS worms could not swim as well as wtFUS or non-transgenic control animals, and this was apparent over a mere matter of hours (Figure 1E). Therefore, this phenotype could easily be used for suppressor screening.

Neuronal dysfunction precedes neurodegeneration in many neurodegenerative diseases (Saxena & Caroni, 2011). To determine if the progressive paralysis phenotypes seen in mFUS animals correlates with motor neuron degeneration, we crossed our transgenic animals with a reporter expressing the fluorescent protein mCherry in the GABAergic neurons (*unc-47p*::mCherry) (McIntire *et al.*, 1997). Similarly, to our previous model of FUS toxicity (Vaccaro *et al.*, 2012), we observed gaps/breaks along the ventral nerve cord in mFUS transgenic animals when compared to worms expressing wtFUS (Figure 1F). We scored neuronal breaks in wtFUS and mFUS animals at day 1, 5 and 9 of adulthood and saw that like paralysis, neurodegeneration was age-dependent and occurred at a higher rate in mFUS animals compared to wtFUS worms (Figure 1G). In addition to the ventral degeneration, we quantified the dorsal axonal degeneration of the GABAergic motor neurons (Figure 1H). We observed age dependant dorsal degeneration occurring in the mFUS worms (Figure 1I), indicating that distal axonal degeneration is occurring in the mFUS strain. Detailed scoring of either ventral, dorsal or both dorso-ventral degeneration at day 9 of adulthood shows that several types of degeneration are more prevalent in the mFUS worm population compared to either wtFUS or *unc-47p*::mCherry control worms (Figure 1J). These results confirm that our single-copy transgenics recapitulate the motor degeneration phenotypes observed in ALS patients.

Here we introduce a novel *C. elegans* model for investigating the mechanisms of motor neuron toxicity in ALS caused by the expression of a single mFUS transgene. Interestingly, although we drove the expression of the human FUS transgenic only in a small subset of neurons (26 out of 302), the expression of the single-copy mFUS was sufficient to phenocopy our multiple-copy human FUS model across all assays. Both models display early neuronal dysfunction and hyperexcitability, which is followed by adult-onset paralysis and neurodegeneration. Therefore, our single-copy mFUS model recapitulate key aspects of the human disease. Furthermore, by simply placing the nematodes into a liquid media, we are able to accelerate the onset of movement phenotypes from a few days into hours. Therefore, our transgenics offer the possibility of high-throughput phenotype-based screening for new therapeutics which can then be validated in vertebrate or mammalian systems. Such screening approaches have been gaining in popularity to accelerate the drug discovery process when the mechanisms underlying the disease remain poorly understood.

## Methods

**Nematode strains and worm maintenance**

Worms were handled and cultured according to standard methods (Stiernagle, 2006). All experiments were performed at 20°C.

**Generation of transgenic worms**

cDNA for human wild-type and mutant FUS were obtained from Dr. Guy Rouleau (Montreal Neurological Institute). Transgenes were integrated into the mosSCI site ttTi605 using the mosSCI system. Made by Knudra Transgenics. All strains have been outcrossed 3 times.

**Paralysis assays**

In 3 separate experiments, 30-35 L4 animals by triplicates were picked to standard NGM plates and scored daily for paralysis starting the following day. Animals were counted as paralyzed if they failed to move their body upon prodding with a platinum wire. Worms were considered dead if they failed to move their head and showed no pharyngeal pumping when prodded; dead or lost animals were censored from statistical analyses. All experiments were conducted at 20°C and worms have been transferred every 2 days to avoid progeny.

**Lifespan assays**

All lifespan experiments were handled similarly to paralysis assays. 3 separate experiments of 30-35 worms by triplicate have been performed at 20°C. Worms were counted every second day from day 1 of adulthood until death. Lost animals were censored from statistical analyses; paralyzed worms were not censored and kept until death. Survival curves were produced and compared using the Log-rank (Mantel-Cox) test.

**Liquid culture motility assays**

Synchronized day 1 adult animals were transferred into 100 uL of M9 buffer in a 96 well plate, to a number of 30 animals per well. The 96 well plates were put into a PhylumTech WMicrotracker-One for automated data analysis over a period of 10 hours.

**Aldicarb sensitivity assays**

To evaluate synaptic transmission, worms were grown on standard NGM plates until day 1 of adulthood, when they were transferred to NGM plates containing 1 mM aldicarb. Worm paralysis was assayed every 30 minutes for 2 hours. Animals were counted as paralyzed if they failed to move upon prodding with a platinum wire.

**Fluorescence microscopy**

Assays were carried out on a Zeiss Axio Observer inverted fluorescence microscope. Worms were immobilized in 5 mM levamisole and mounted on 2% agarose pads. For neurodegeneration assays, scoring of axonal breaks was performed at day 1, 5 and 9 on live animals for GABAergic motor neurons. Around 100 animals were scored per condition or genotype over 4 trials. Degenerative events were classified as ventral or dorsal, depending on their location on either the ventral or dorsal part of the worm. Worms showing degenerative events on both sides were classified as ventro-dorsal degeneration.

**RNA extraction and TAQMan assay**

Total RNA was obtained from *C. elegans* using the “RNA extraction for RNA-Seq” protocol from the Bowdish Lab (McMaster University, Hamilton, Canada), quantified photometrically with a NanoPhotometer (Implen) and stored at -80°C until further use. For gene expression analysis, cDNA from 1500 ng total RNA was generated using the Superscript Vilo IV cDNA synthesis kit (Thermo Fischer Scientific). Samples were used, undiluted, and yielded a CT value between 15 and 35. Gene expression was analyzed using TaqMan Gene Expression Assays (Applied Biosystems) and a QuantStudio 3 Real-Time PCR System (Thermo Fisher). Data were normalized to the housekeeping gene *ama-1* and analyzed using the Δ/Δ-CT method. Undetermined CT values (below detection) were considered with a CT value of 40 (maximum cycle for the run) for the purpose of the Δ/Δ-CT analysis. All experiments were made four times.

**Statistical analyses**

Paralysis curves were generated and compared using the log-rank (Mantel–Cox) test. All experiments were repeated at least three times. For neurodegeneration assays, the mean was calculated for each trial and analyzed by unpaired t-tests. For TAQMan assay, the data was analyzed using the Δ/Δ-CT method for each trial and analyzed by one-way ANOVA. Quantitative data were expressed as mean ± SEM. GraphPad Prism v8 software was used for all statistical analyses.

## Reagents

**Table:** 

**Strain**	**Genotype**	**Available from**
N2	Wild type C. elegans strain	CGC
CB307	*unc-47(e307)*	CGC
LX929	*vsIs48[unc-17::GFP]*	CGC
IZ629	*ufIs34[Punc-47p::mCherry]*	Kind gift from from Dr. Michael M. Francis (University of Massachusetts, Worcester, MA)
AP18.1	*knuSi499 [ AP18 (FUS^WT^, unc-119(+) ) ] II ; unc-119(ed3) III*	Parker Lab
AP19.1	*knuSi498 [ AP19 (FUS^S57Δ^, unc-119(+) ) ] II ; unc-119(ed3) III*	Parker Lab
XQ471	FUS^WT^*;unc-17::GFP;unc-47::mcherry*	Parker Lab
XQ472	FUS^S57Δ^*;unc-17::GFP;unc-47::mcherry*	Parker Lab
		
**TAQMan probe**	**Gene**	**Available from**
Hs01100224_m1	Human *FUS*	Thermo Fisher Scientific
Ce02462726_m1	*ama-1*	Thermo Fisher Scientific

## References

[R1] Belzil VV, Valdmanis PN, Dion PA, Daoud H, Kabashi E, Noreau A, Gauthier J, Hince P, Desjarlais A, Bouchard JP, Lacomblez L, Salachas F, Pradat PF, Camu W, Meininger V, Dupré N, Rouleau GA, S2D team. (2009). Mutations in FUS cause FALS and SALS in French and French Canadian populations.. Neurology.

[R2] Conte A, Lattante S, Zollino M, Marangi G, Luigetti M, Del Grande A, Servidei S, Trombetta F, Sabatelli M (2011). P525L FUS mutation is consistently associated with a severe form of juvenile amyotrophic lateral sclerosis.. Neuromuscul Disord.

[R3] Frøkjær-Jensen C, Davis MW, Ailion M, Jorgensen EM (2012). Improved Mos1-mediated transgenesis in C. elegans.. Nat Methods.

[R4] Mahoney TR, Luo S, Nonet ML (2006). Analysis of synaptic transmission in Caenorhabditis elegans using an aldicarb-sensitivity assay.. Nat Protoc.

[R5] McIntire SL, Reimer RJ, Schuske K, Edwards RH, Jorgensen EM (1997). Identification and characterization of the vesicular GABA transporter.. Nature.

[R6] Renton AE, Chiò A, Traynor BJ (2013). State of play in amyotrophic lateral sclerosis genetics.. Nat Neurosci.

[R7] Saxena S, Caroni P (2011). Selective neuronal vulnerability in neurodegenerative diseases: from stressor thresholds to degeneration.. Neuron.

[R8] Schmeisser K, Fardghassemi Y, Parker JA (2017). A rapid chemical-genetic screen utilizing impaired movement phenotypes in C. elegans: Input into genetics of neurodevelopmental disorders.. Exp Neurol.

[R9] Shang Y, Huang EJ (2016). Mechanisms of FUS mutations in familial amyotrophic lateral sclerosis.. Brain Res.

[R10] Stiernagle T (2006). Maintenance of C. elegans.. WormBook.

[R11] Vaccaro A, Tauffenberger A, Aggad D, Rouleau G, Drapeau P, Parker JA (2012). Mutant TDP-43 and FUS cause age-dependent paralysis and neurodegeneration in C. elegans.. PLoS One.

[R12] Vashlishan AB, Madison JM, Dybbs M, Bai J, Sieburth D, Ch'ng Q, Tavazoie M, Kaplan JM (2008). An RNAi screen identifies genes that regulate GABA synapses.. Neuron.

[R13] Vucic S, Rothstein JD, Kiernan MC (2014). Advances in treating amyotrophic lateral sclerosis: insights from pathophysiological studies.. Trends Neurosci.

